# Spatial and Temporal Variation in the Effects of Climatic Variables on Dugong Calf Production

**DOI:** 10.1371/journal.pone.0155675

**Published:** 2016-06-29

**Authors:** Mariana M. P. B. Fuentes, Steven Delean, Jillian Grayson, Sally Lavender, Murray Logan, Helene Marsh

**Affiliations:** 1 College of Marine and Environmental Sciences, James Cook University, Townsville, Queensland, Australia; 2 Department of Earth, Ocean and Atmospheric Science, Florida State University, Tallahassee, Florida, United States of America; 3 School of Biological Sciences and The Environment Institute, The University of Adelaide, Adelaide, South Australia, Australia; 4 CSIRO Oceans and Atmosphere Flagship, Aspendale, Victoria, Australia; 5 Australian Institute of Marine Science, Townsville, Queensland, Australia; 6 Tropical Water and Aquatic Ecosystem Research, James Cook University, Townsville, Queensland, Australia; New York Institute of Technology College of Osteopathic Medicine, UNITED STATES

## Abstract

Knowledge of the relationships between environmental forcing and demographic parameters is important for predicting responses from climatic changes and to manage populations effectively. We explore the relationships between the proportion of sea cows (*Dugong dugon)* classified as calves and four climatic drivers (rainfall anomaly, Southern Oscillation El Niño Index [SOI], NINO 3.4 sea surface temperature index, and number of tropical cyclones) at a range of spatially distinct locations in Queensland, Australia, a region with relatively high dugong density. Dugong and calf data were obtained from standardized aerial surveys conducted along the study region. A range of lagged versions of each of the focal climatic drivers (1 to 4 years) were included in a global model containing the proportion of calves in each population crossed with each of the lagged versions of the climatic drivers to explore relationships. The relative influence of each predictor was estimated via Gibbs variable selection. The relationships between the proportion of dependent calves and the climatic drivers varied spatially and temporally, with climatic drivers influencing calf counts at sub-regional scales. Thus we recommend that the assessment of and management response to indirect climatic threats on dugongs should also occur at sub-regional scales.

## Introduction

Understanding the mechanisms underpinning population dynamics is central to many ecological and evolutionary questions and the development of effective conservation strategies [1–3]. The dynamics of a population are a function of its key demographic parameters such as mortality, fecundity and migration rates [4]. All these parameters are directly and/or indirectly influenced by environmental and climatic drivers (e.g., [5–7]). Indirect pathways occur mainly through reductions in food and the availability of quality habitat (e.g., [8]).

Interest in the relationship between demographic parameters and environmental and climatic drivers has increased as a result of concerns about the ecological impacts of climate change on individual species (e.g., [9, 10]). Understanding the relationship between environmental forcing and demographic parameters is an important first step in predicting the impacts of extreme weather events and climate change [5, 11]. For example, understanding the relationship between the number of breeding green turtles, *Chelonia mydas*, in Australia and the Southern Oscillation Index (SOI) has enabled the annual green turtle nesting population at key eastern Australian rookeries to be predicted with reasonable confidence based on the SOI, two years before the commencement of a breeding season [8, 12]. The SOI is an index of the El Niño phenomenon based on the difference in pressure between Tahiti and Darwin, in northern Australia. This relationship suggests that the El Niño, an ocean-basin scale climatic driver, influences the proportion of sub-adult and adult females able to acquire the fat reserves necessary to breed, a proportion limited by the availability of their food, principally seagrass and algae [8]. Mass nesting is generally recorded two years after major El Niño events, while extremely low nesting numbers tend to be recorded two years after major La Niña events [8]. This knowledge is potentially of great value for managing green turtles in the Australian region, particularly in areas where nesting females and their eggs are harvested [12].

Environmental and climatic drivers also influence key demographic parameters of another herbivore dependent on seagrass communities, the dugong, *Dugong dugon*, commonly knows as the sea cow [13, 14]. Dugongs occur sympatrically with green turtles in the coastal and island waters of the tropical and Indo-West Pacific [5]. Extreme weather events (e.g., cyclones and flooding) have been associated with the following impacts on dugongs: mass stranding, increased movements presumably in search of food, loss of weight and fat, delayed reproduction and mortality [13–17]. For example, the proportion of dependent calves in the dugong population in Hervey Bay (Queensland, Australia) plummeted after two floods and a cyclone in 1992, a sequence of extreme weather events that caused the loss of more than 1000 km^2^ of seagrass in the region [14].

The urbanized coast of Queensland, Australia spans some 12° latitude. In the summer of 2010/11, this region was severely impacted by extreme weather events including the strongest La Niña weather pattern since 1973, major floods and three tropical cyclones. These events followed several years of deterioration in some seagrass communities as a result of unusually wet weather [18–20]. Dugongs moved from affected areas, suffered record mortality [5], and a reduction in fecundity and neonatal survivorship was observed as evidenced by the proportion of dugongs classified as dependent calves [13, 21]. Aerial surveys following these extreme weather events suggested that the dugongs’ responses were geographically variable and much more evident in the Southern Great Barrier Reef sub-region (latitudes 15° 30’ S to 24° 30’S) than in Hervey Bay (25°17′ S) or Moreton Bay (27° 28’S) [21]. These examples suggest that the dugong’s demographic parameters can be negatively impacted by key climatic drivers at sub-regional (100s km) latitudinal scales.

We investigated how the proportions of dugong calves recorded during a time series of standardized aerial surveys since the 1970s were associated with various sub-regional and ocean-basin climatic covariates at a range of spatially distinct sub-regions along the east coast of Queensland Australia, an area with relatively high dugong density.

## Materials and Methods

The aerial surveys were conducted under permit from the Great Barrier Reef Marine Park Authority and the Queensland environment department. Dugong research at James Cook University was conducted under the permits issued by the JCU Animal Ethics Committee.

### Study Region

**Fig 1:**
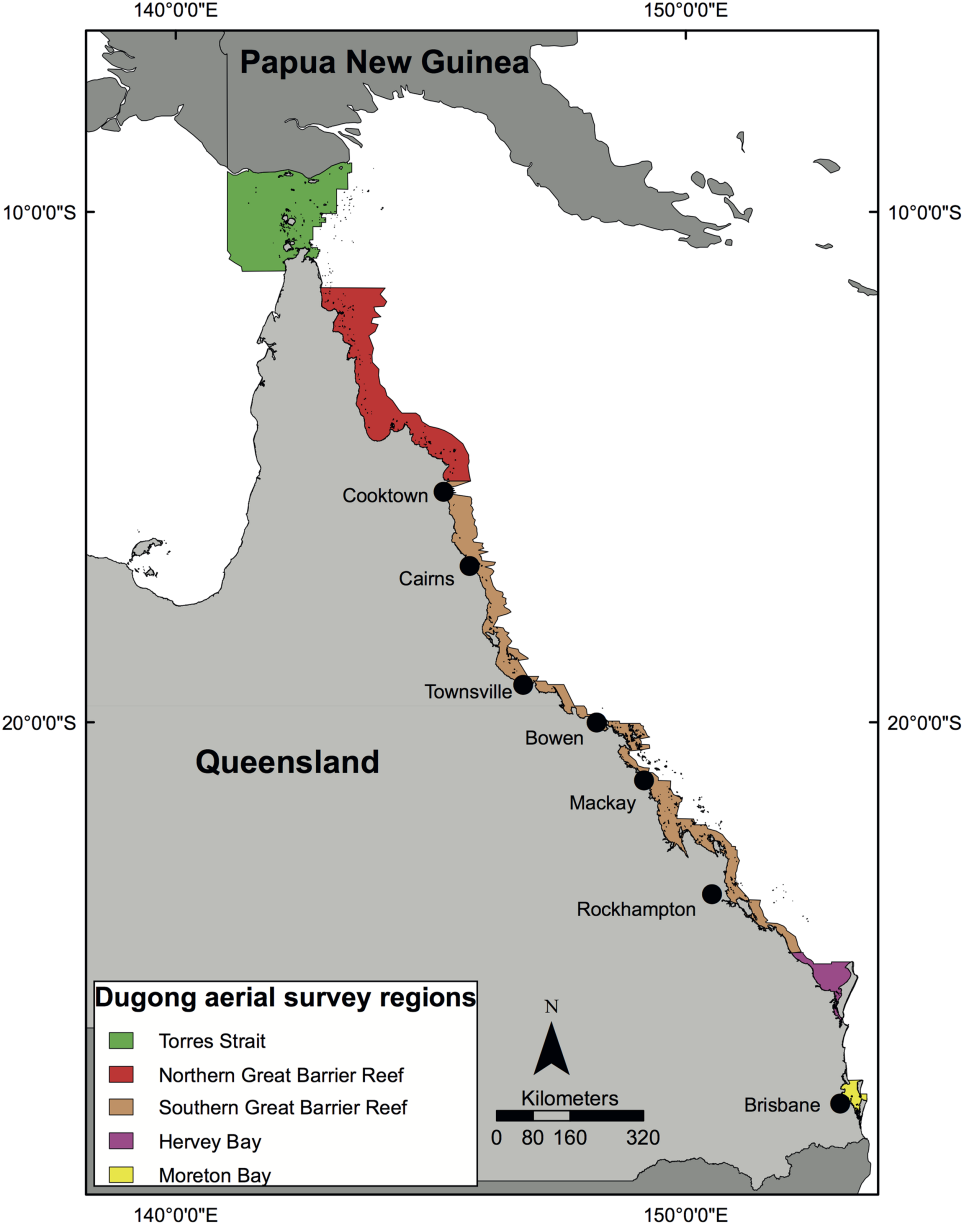
The five dugong aerial survey sub-regions along the each coast of Queensland. This figure is reproduced with permission from Fig 1 in Grech et al. [2011) Informing Species Conservation at Multiple Scales Using Data Collected for Marine Mammal Stock Assessments. PLoS ONE 6(3): e17993. doi:10.1371/journal.pone.0017993.

Our study area was the waters of the eastern Queensland coast (Fig 1), including Torres Strait. This region supports globally significant populations of dugongs [13]. We divided the coast into 5 sub-regions (Torres Strait, Northern and Southern Great Barrier Reef (GBR), Hervey Bay and Moreton Bay) (Fig 1) to match the biological datasets used for this study (Table 1).

**Table 1:** Details of the aerial survey data used in this paper. All data collected from 1984 are on-line at https://dugongs.tropicaldatahub.org.

Subregions	Geographical details of subregion	Survey years
**Torres Strait**	10° 29’S 142° 10’ E; 29 764 km^2^	1991, 1996, 2001, 2006, 2011, 2013
**Northern Great Barrier Reef**	11°32’S -15°30’S; 20132 km^2^	1978, 1984, 1985, 1990, 1995, 2000, 2006, 2013
**Southern Great Barrier Reef**	15°30’S-24° 30’S; 33676 km^2^	1974, 1975, 1976, 1977, 1978, 1979, 1986, 1992, 1994, 1999, 2005, 2011
**Hervey Bay**	25° 17′ S; 6156 km^2^	1979, 1988, 1992, 1993, 1994, 1999, 2001*, 2005, 2011
**Moreton Bay**	27° 28' S; 2192 km^2^	1976, 1977, 1979, 1995, 1999, 2000, 2001*, 2005, 2011

*Surveys conducted both in autumn (April) and summer (November).

### Calf counts

Standardized aerial surveys have been conducted in eastern Queensland by our James Cook University (JCU) group since the middle of the 1970s (Table 1). The aerial survey methodology used the techniques detailed in Marsh (1981), Marsh and Sinclair (1989a) and Marsh et al. (2015) [22–24]. The surveys were conducted by flying systematically placed transects at heights of 450–900 feet (137 m) above sea level over defined survey regions at spatial scales of thousands of km^2^, with the aim of obtaining robust estimates of dugong relative density and abundance (see for examples [14, 22, 25–29]). The experimental work of Marsh and Sinclair (1989b) [30] indicates that there should be no difference in dugong sightability between the survey heights used.

The quality control over the aerial surveys was very high. There was a strict ceiling on survey conditions. Trained teams of two observers on each side of the aircraft scanned the defined transects and recorded sightings onto separate tracks of an audio-recorder enabling post-survey calibration of observer reliability. Group sizes were small (mode 1 or 2). Dependent calves were recorded explicitly for each sighting, enabling the proportion of dugongs that were dependant calves to be calculated for each survey. A calf was identified as an animal swimming in close proximity to another dugong and less than two thirds of the size of that animal. Calves were reliably distinguished by the trained observers as verified by comparing the records of tandem observers post-survey. As no defined calving or nursery areas have been identified for dugongs in this region, the proportion of calves recorded should reflect actual differences between surveys rather than differences between observers or survey conditions.

Marsh et al. [14] summarised the literature on dugong reproduction. Gestation is estimated to last about 14 months and calves are dependent on their mother for some 18 months, even though they start eating seagrass soon after birth. The animals classified as calves during aerial surveys are aged from neonates to about 18 months. Thus the proportion of dependant calves sighted during an aerial survey (calf production) is a reflection of births (which are expected to reflect the effect of environmental conditions over several years on female fecundity) and neonatal survivorship (which can be affected by the more immediate effect of an extreme weather event on the mortality of both mothers and calves as a result of mass stranding associated with a storm surge as well as starvation due to loss of seagrass beds).

The sampling effort reflected the availability of resources (see Table 1). The sub-regions were chosen for logistical and jurisdictional reasons and were generally separated by tracts of coast in which dugong density is low. Aerial surveys were conducted mostly during late spring or early summer when weather and sea states provide optimum survey conditions. However, since dugongs move in response to seasonal changes in water temperatures at the higher latitudinal limits to the dugong’s range in Moreton Bay and Hervey Bay [31] some aerial surveys in these sub-regions were also conducted during autumn (Table 1). Given the dugong’s diffusely seasonal breeding cycle and protracted period of calf dependency [13, 32], seasonal differences in the timing of aerial surveys should not have affected the proportion of dugongs classified as calves.

### Climatic covariates

The influences of the following climatic covariates on the proportion of dependent calves in a sub-region were assessed: 1) rainfall anomaly—wet season rainfall anomaly (difference between wet season rainfall for a surveyed subregion and the 30 year average for that subregion; 2) SOI—the November to February Southern Oscillation Index (SOI) based on the difference in pressure between Tahiti and Darwin; 3) NINO 3.4—the November to February NINO 3.4 sea surface temperature index based on sea surface temperature in the NINO 3.4 region of the equatorial Pacific Ocean; and 4) number of tropical cyclones (TC). These covariates are highly correlated. SOI and Nino 3.4 are both indices of the El Niño-Southern Oscillation phenomenon. There is also a strong relationship with eastern Australian tropical cyclone impacts and the El Niño-Southern Oscillation phenomenon, with almost twice as many impacts during La Niña than during El Niño (Australian Bureau of Meteorology http://www.bom.gov.au/cyclone/about/eastern.shtml).

Data on the first three climatic covariates were obtained from the Australian Bureau of Meteorology (http://www.bom.gov.au/). The cyclone track data were obtained from the International Best Track Archive for Climate Stewardship (IBTrACS) dataset [33]. Tropical cyclone tracks were interpolated onto a 1°× 1° grid and the area of influence of each tropical cyclone was calculated using an effective radius of 5° longitude/latitude. A tropical cyclone was included in the analysis if it came within 5° of the coastline of any of the sub-regions in Table 1.

### Data analysis

#### Exploratory analysis

The data were explored for over-dispersion by overlaying the proportion of dependent calves per subregion and year with the probability from a null Binomial generalised linear model. The dataset was also explored for zero-inflation by comparing the percentage of zeroes in the data to the percentage expected from a binomial distribution with a centrality parameter equal to that estimated from the data.

#### Data processing

The initial exploratory data analysis suggested that the data were over-dispersed (relative to a Binomial distribution), yet not zero-inflated. Hence all models were fitted against a Beta-Binomial (logit) distribution. Temporal trends in the proportion of dugongs with calves were estimated using via a Beta-Binomial (logit-link), Bayesian penalized spline regression [34]:

$$\begin{array}{l} y_{ij}\;\sim \;Binomial\left(\pi_{ij},\;n_{ij}\right)\\ \pi_{ij}\sim \;Beta\left(a_{ij},\;b_{ij}\right)\\ a_{ij}=\;\theta \pi_{ij}\\ b_{ij}=\;\theta \left(1-\pi \right)\\ logit\left(\pi_{ij}\right)=f\left(t_{ij}\right)+f_{l}\left(t_{ij}\right)\\ \{\begin{array}{c} f\left(t\right)=\;\beta_{0}+\;\beta_{1}t+\Sigma_{k=1}^{K}r_{k}Z_{tk}\\ f_{i}\left(t\right)=\;\gamma_{0_{l}}I_{\left(l>1\right)}+\;\gamma_{1_{t}}I_{\left(l>1\right)}+\;\Sigma_{k=1}^{K}s_{lk}z_{tk} \end{array}\\ \beta_{0},\beta_{1}\sim \;N\left(0,\;\sigma^{2}\right)\\ \gamma_{0_{l}},\;\gamma_{1_{l}}\sim \;N\left(0,\sigma_{\gamma }^{2}\right),\;l=1\ldots .5\\ r_{k}\sim \;N\left(0,\;\sigma_{r}^{2}\right),\;k=1\ldots .K\\ s_{lk}\sim \;N\left(0,\;\sigma_{s}^{2}\right),\;l=1\ldots .5,\;k=1\ldots .K\\ 0,\;\theta \;\sim \;Unif\left(0,100\right) \end{array}$$

where n_ij,_ *t*_ij and_ y_ij,_ are the number of calves, the total number of dugongs and the year within sub-region _*i*_ and year _*j*_ respectively. f(*t) and* f_l_ (*t*) respectively represent the overall smooth curve and the deviations of each of the *l* sub-regional dugong populations from this overall curve, r_*k*_ represents the sub-region random effects and accounts for the spatial autocorrelation structure of the model.

z_tk_ represents a design matrix for the thin-plate spline with three knots (*K* = 3). Non-informative normal priors were specified for all model parameters (β_0_, β_1_, **γ**_0_, **γ**_1_,***δ***^2^, s) and weekly informative half-cauchy (scale = 25) priors were specified for variances [35].

To explore the temporal trends, 1500 samples were collected from three chains with a total of 300,000 iterations, burnin of 50,000 per chain and thinning rate of 10. Chain mixing and convergence were assessed via traceplots, autocorrelation and Gelman-Rubin diagnostics (all scale reduction factors less than 1.05).

As dugongs have a long reproductive cycle [13, 36], calf production is likely to be impacted by past conditions, with each climatic covariate potentially influencing calf counts with a lagged and/or instantaneous effect [5]. Therefore, a range of lagged versions of each of the focal climatic covariates, scaled to a mean of 0 and standard deviation of 1, were all included in a global model containing proportion of calves in each population crossed with each of the lagged versions of the covariate. The relative influence of each predictor was estimated via Gibbs variable selection [37]. For each sample of the Markov chain Monte Carlo (MCMC), parameters associated with each covariate were included with probabilities defined by associated Bernoulli distributed weightings from which Gibbs variable selection was used to determine the relative influence of each predictor on the model. This approach also accounted for the correlations between the covariates explained above. All Bayesian models were fitted using JAGS [38] using the R2jags [39] and coda [40] packages for R [41]. Covariates with posterior model probabilities exceeding 0.5 (50% of models) were considered important predictors of the proportion of dependent calves in a population and their influences are illustrated.

## Results

### Proportion of calves

**Fig 2:**
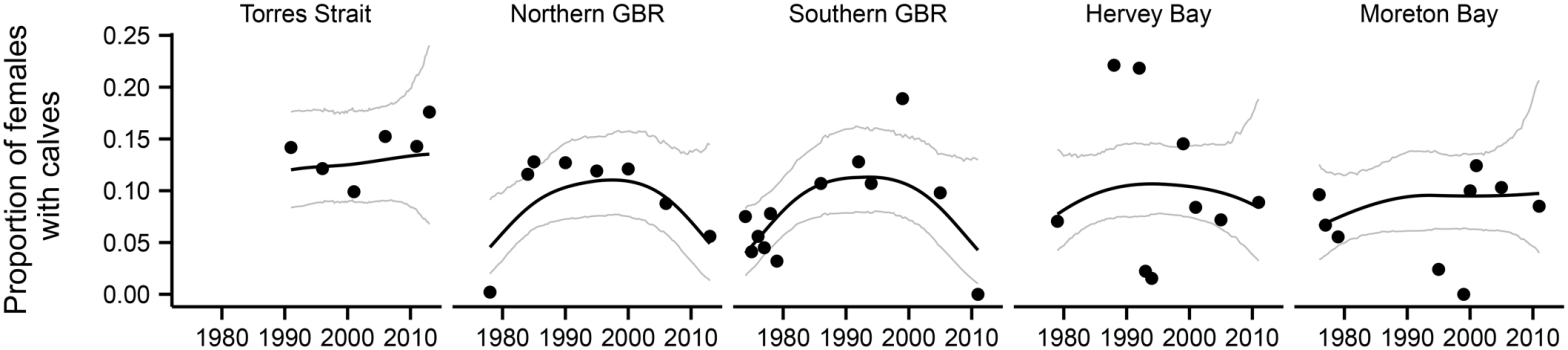
Trends in proportion of calves including linear smoothers for each sub-region across the study period (1974–2013). Trends in SOI and Nino 3.4 for the same period.

The proportion of calves varied across the different sub-regions and surveys (Table 2 and Fig 2) ranging from 0 in Moreton Bay in 1999 and the Southern Great Barrier Reef in 2011 to 0.22 in Hervey Bay in 1988 and 1992. The average proportion of calves over all of the years in each location ranged from 0.072 in Moreton Bay to 0.139 in Torres Strait (Table 2). The temporal changes in the proportion of calves were inconsistent across sub-regions. For example, the proportion of calves increased in the Northern and Southern Great Barrier Reef regions in the late 1990s before declining (Fig 2), while the proportion of dugongs with calves increased in Torres Strait post 2000.

**Table 2:** Proportion of dugong calves for each subregion during the study period.

Subregion*	# years*	Average proportion of calves (range)
**Torres Strait**	6	0.139 (0.099–0.176)
**Northern Great Barrier Reef**	8	0.094 (0.002–0.128)
**Southern Great Barrier Reef**	12	0.079 (0–0.188)
**Hervey Bay**	9	0.104 (0.015–0.221)
**Moreton Bay**	9	0.072 (0–0.124)

*For details of regions and survey years refer to Table 1.

### Spatial and temporal variation in the effect of climatic covariates on fecundity

**Fig 3:**
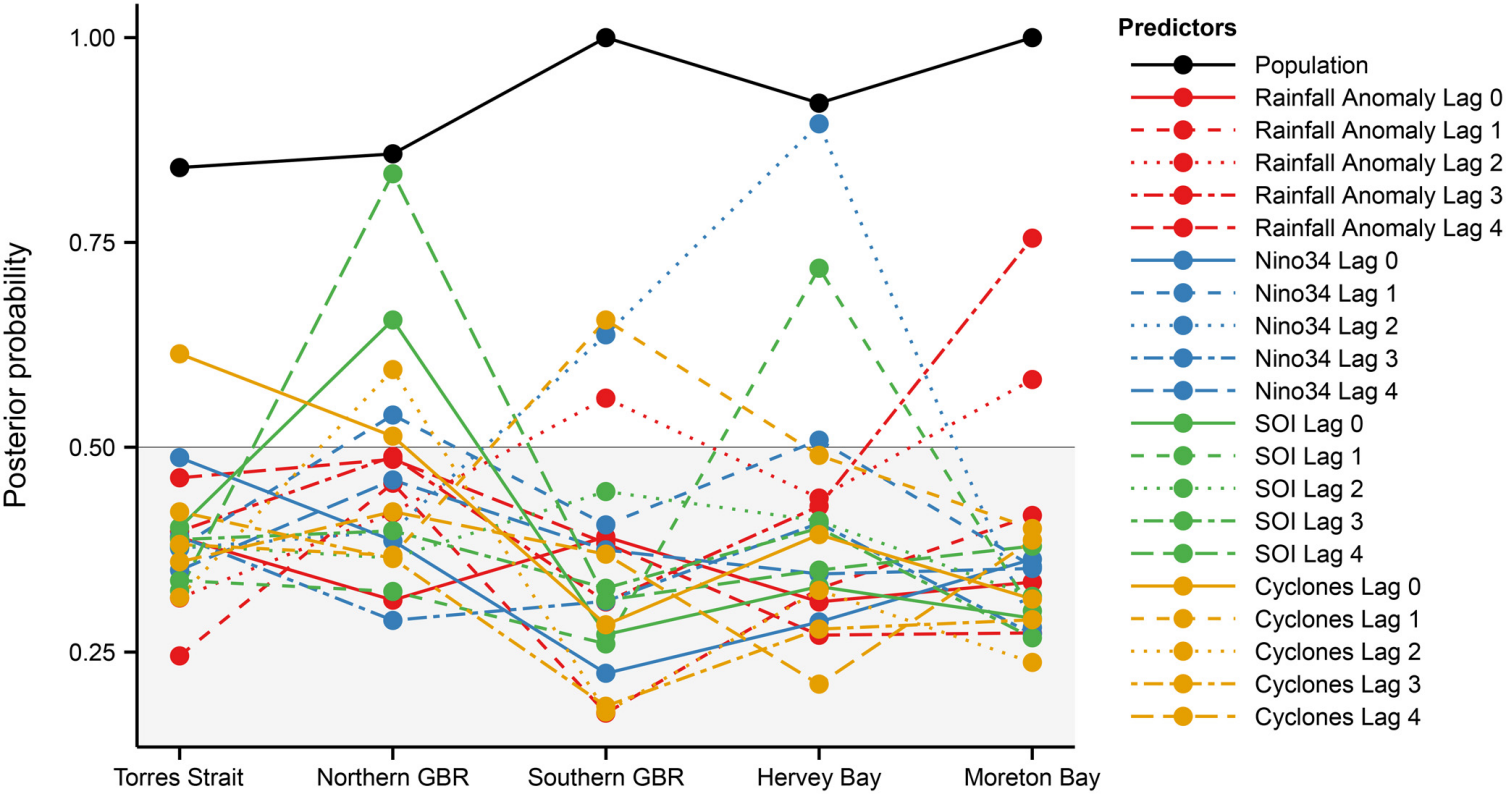
Gibbs variable selection posterior model probabilities for Beta-Binomial model including population crossed with various lagged and scaled environmental covariates. The higher the posterior probability, the more often the term was included in the model. Variables with posterior model probabilities exceeding 0.5 (50% of models) were considered important predictors of the proportion of dugong calves and are illustrated here.

**Fig 4:**
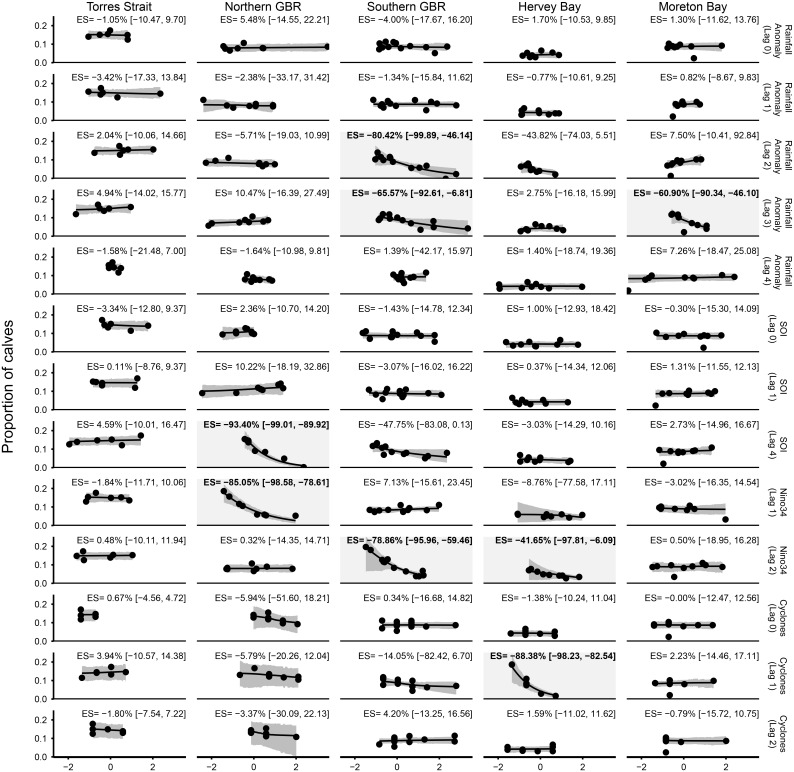
Partial effects in the global model of the climatic covariates for which Gibbs predictor was > 0.5 on the proportion of dugong sighted that were calves on aerial surveys (x axis). The y axes are scaled to mean of zero and standard distribution of 1. The significant effects (95% Credibility Interval of % Effect Size (ES) does not include 0) are shaded.

The influence of each climatic covariate on the proportion of calves and the associated time lags varied spatially and temporally (Figs 3 and 4). The proportion of calves in Torres Strait did not vary with changes in cyclone numbers or any climatic covariate (Fig 4). In the Northern Great Barrier Reef region, the proportion of calves declined in association with the increase in both the SOI (lagged to four years) and Nino 3.4 (lagged to 1 year; Fig 4). In the Southern Great Barrier Reef, the proportion of calves declined with: 1) increasing rainfall above the long-term average (lagged to 2 and 3 years); and 2) increases in Nino 3.4 (lagged 2 years; Fig 4). In Hervey Bay, the proportion of calves declined with increases in Nino 3.4 (lagged 2 years) and the number of tropical cyclones (lagged 1 year; Fig 4). In Moreton Bay the proportion of calves declined with increasing rainfall above the long-term average (lagged to 3 years; Fig 4).

## Discussion

The climatic drivers we tested influenced the proportion of dugongs classified as calves at sub-regional scales. The proportion of calves was negatively correlated with various features of La Niña episodes (lagged high SOI—above average rainfall, cyclones) in the Northern and Southern Great Barrier Reef, Hervey Bay and Moreton Bay, even though the response differed between the sub-regions. This response pattern presumably reflects the declining status of seagrass associated with these climatic variables [14, 18, 19]. Calf counts were also negatively correlated with lagged Nino3.4 in the Northern and Southern Great Barrier Reef, a feature of the El Niño phase based on sea surface temperature, which likely affects seagrass beds directly. The photosynthetic condition of eastern Australian sub-tropical and tropical seagrass species can suffer irreparable effects from short-term or episodic changes in seawater temperatures as high as 40–45°C, with variability in species tolerance to thermal stress [42]. Increased duration (more days in a row) of thermal events above 40°C is also likely to affect the ecological function of tropical seagrass meadows [43]. Acute stress responses of seagrasses to elevated seawater temperatures are consistent with observed reductions in above-ground biomass during an El Niño events [42]. The differences between the response to the Nino 3.4 climatic variable and the different locations likely reflects the species composition and depth profile of the seagrass beds in the different sub-regions.

The climatic covariates that were significantly associated with the proportion of dependent calves in a dugong population (Fig 4) were always lagged by at least one year. The variation in the duration of the lags may be an artefact of the timing of the aerial surveys, which was independent of the pattern of climatic variation. Nonetheless the fact that the significant covariates were always lagged, presumably reflects the need for dugongs to be in good condition prior to and during the prolonged period of pregnancy and lactation [17]. Thus, dugong calf counts appear to be more influenced by the condition of the mother several years prior to a survey rather than the more immediate impacts of climatic drivers and storms on dugongs [13–16] and their seagrass habitats [14, 18, 19]. This result presumably reflects the fact that the influence of unlagged SOI, elevated freshwater discharge and low air temperatures [5] increases the mortality of both adult dugongs and their calves, along the Queensland coast and thus does not affect the proportion of calves *per se*.

Dugong mortality is also affected by finer scale climatic drivers [5]. Across the urbanized Queensland coast (from Cairns 16° 55’ S to the NSW border 28° 10’ S; Fig 1), dugong mortality was predicted by sustained periods of elevated freshwater discharge and low air temperatures [5], conditions associated with La Niña episodes. However, when analyses were conducted for specific latitudinal areas (e.g., Townsville, Moreton Bay and Hervey Bay) there were differences. These results suggest that: 1) research on the influence of climatic drivers on the demography of the dugong needs to be conducted at sub-regional spatial scales, and 2) predictive models of the impacts of extreme weather events and climate change on dugongs also need to be developed at sub-regional scales.

### Spatial variation in the effect of climatic covariates on the proportion of dependent calves

The relationships between the proportion of dependent calves and climatic drivers varied spatially and temporally. In the Torres Strait, which is the most important dugong habitat in the world [14] and is one of the tropical coastal areas in the world least impacted by humans [44], the dugong population, presented lower variation in the proportion of dependent calves than in any other region (Fig 2) and this proportion was not associated with any of the climatic covariates explored here. Torres Strait lies north of the main cyclone belt of the Great Barrier Reef, and is thus less prone to severe tropical storms than the Great Barrier Reef coast [45, 46]. Nonetheless, the extensive seagrass meadows in Torres Strait are known to disappear episodically over broad areas [47, 48]. The 1970s seagrass loss, which occurred before the dugong surveys reported here, was associated with changes in the dugong pregnancy rate as recorded by carcass analysis [14]. The causes of such episodic seagrass losses are unknown. Modelling indicates that neither the turbidity from the rivers on the south coast of Papua New Guinea [49] nor sand dune crest migration [47] are likely to have affected the seagrass communities in Torres Strait at the scale of the seagrass losses.

In the other sub-regions studied here, the proportions of calves sighted in the aerial surveys were variably associated with the climatic covariates we tested. In the Northern Great Barrier Reef, a very significant dugong habitat [14], that is generally considered to be in better condition that the inshore waters of the southern Great Barrier Reef [50], dugong calf counts were negatively associated with the La Niña phenomenon (SOI lagged 4; Fig 4), which is usually associated with above average rainfall and cyclonic activity. The Northern GBR sub-region is in the cyclone belt and often affected by cyclones (http://www.bom.gov.au/cyclone/about/eastern.shtml#history). The proportion of calves also declined in association with the Nino 3.4 Index (lagged to 1 year; Fig 4).

The Southern GBR sub-region, which is also in the cyclone belt (http://www.bom.gov.au/climate/maps/averages/tropical-cyclones/), is subjected to greater human impacts than the Northern Great Barrier Reef sub-region [50]. Calf counts were negatively associated with heavy rainfall lagged by 2 and 3 years and the Nino3.4 lagged by 2 years (Fig 4), suggesting that multiple drivers were affecting the dugong’s food supply, a result consistent with the findings of Meager and Limpus [5]. Responses to the extreme weather events of 2010/11 in the eastern Queensland coast, Australia, including the strongest La Niña weather pattern since 1973, major floods and three cyclones, were most evident in the Southern Great Barrier Reef sub-region, where no calves were seen during an aerial survey in late 2011 [22]. Seagrass was in poor condition in the southern Great Barrier Reef even prior to the extreme weather events of 2011 [18, 20].

The influence of extreme weather events on the proportion of dependent calves in a dugong population was also obvious in Hervey Bay, where there was a strong negative effect of the number of tropical cyclones lagged by 1 year (Fig 4) and increases in Nino 3.4 (lagged 2 years; Fig 4). Nonetheless the response was variable. The proportion of dugong calves in Hervey Bay declined from 22% to 2.2% in a year following two floods and a cyclone in 1992, which caused the loss of more than 1000 km^2^ of seagrass in the region [14], but was within normal range after the extreme weather events of 2010/11 [21], likely reflecting the recent history of seagrass condition in this region.

Despite Moreton Bay being adjacent to the major city of Brisbane, the important dugong habitat in the eastern bay has a relatively low level of anthropogenic impact due to its physical separation from the main terrestrial interface with the Queensland coast and daily flushing regime with ocean waters [51]. Moreton Bay is also south of the main cyclone belt on the east coast of Australian (http://www.bom.gov.au/climate/maps/averages/tropical-cyclones/) and does not have a history of large natural physical disturbance such as storms and cyclones [51]. In contrast to the Great Barrier Reef sub-regions, especially the southern Great Barrier Reef, calf counts in Moreton Bay were negatively influenced by abnormally heavy rainfall lagged by 3 years. Nonetheless, the calf counts in 2011 and 2013 after the major Brisbane River floods of 2011 were within normal range (Fig 2 and unpublished data).

### Implications for management

Dugongs are long-lived slow breeding animals and the greatest influence on their population dynamics is adult survivorship [13]. Hence, dugong conservation management has focused on direct threats (e.g. bycatch, Indigenous harvest, vessel strike). Nonetheless the indirect effect of freshwater discharge and low water temperatures on dugong mortality has been demonstrated [5]. Our analysis supports the need for managers to consider the effects of indirect stressors (e.g., habitat degradation, food availability), which can influence dugong population dynamics. Managers can address indirect threats mostly through habitat protection and proper coastal management, which will help ensure that seagrass meadows are healthy [13, 52]. Significant loss of seagrasses can also result from extreme rainfall events, tropical storms and La Niña episodes periods, which can result in regional scale decreases in dugong calf production and increases in dugong mortality. These impacts can be expected to last several years. Aerial survey calf counts can be used as a robust index of the status of the dugong population to inform the timing and spatial extent of consequent modification of management responses to direct threats such as bycatch, Indigenous harvest and vessel strike.

Consideration of indirect threats from climatic processes will be even more pressing as climate change progresses and emergency responses become more necessary [53]. Knowledge of the relationship between climatic drivers and demographic parameters and the lag between an event and impact strengthens the need to restrict direct impacts on dugongs and their seagrass habitats to increase their resilience to climatic drivers. Given the spatial variability of the relationships between the proportion of dependent calves in a population and the climatic drivers examined here, we recommend that the assessment and management of indirect climatic threats are conducted at sub-regional scales. It is important, however to remember that environmental factors not considered here may also affect seagrass (e.g., light deprivation from sediment suspension during prolonged periods of high winds [54], toxic algal blooms [55] and that dugong life history parameters may also be influenced by density-dependent responses to changes in population size [17].
